# Effect of sleep on development of early childhood caries: a systematic review

**DOI:** 10.1007/s40368-022-00753-3

**Published:** 2022-09-22

**Authors:** D. Sardana, B. Galland, B. J. Wheeler, C. K. Y. Yiu, M. Ekambaram

**Affiliations:** 1 Division of Pediatric Dentistry, University of Oklahoma College of Dentistry, Oklahoma City, OK USA; 2 Department of Women’s and Children’s Health, Otago Medical School, University of Otago, Dunedin, New Zealand; 3 Division of Pediatric Dentistry, Faculty of Dentistry, The University of Hong Kong, Pok Fu Lam, Hong Kong SAR; 4 Department of Oral Sciences, Faculty of Dentistry, University of Otago, 310 Great King Street, Dunedin, 9016 New Zealand

**Keywords:** Caries, Sleep, Risk factor, Quality of life, Paediatrics, Childhood

## Abstract

**Purpose:**

To investigate the impact of sleep on the development of early childhood caries (ECC).

**Methods:**

Seven electronic databases and grey literature were searched with various keyword combinations. Two reviewers independently selected studies, extracted data, and assessed the risk of bias using the Newcastle–Ottawa Scale. The studies were included if they evaluated the impact of sleep parameters on the caries experience or severity of ECC in children under 6 years of age.

**Results:**

Four cross-sectional studies and two longitudinal studies were included. Children who had irregular bedtimes had a 66–71% higher chance of developing ECC. Children who slept after 11 pm might have a 74–85% higher chance of developing ECC. Children who slept less than 8 h during the night had a 30% increased risk of caries than children who slept more than 11 h.

**Conclusion:**

Irregular or late bedtime and fewer sleeping hours could be an independent risk factor for ECC. The risk of ECC might be related inversely in a dose–response manner to the number of sleep hours.

## Introduction

Sleep problems during early childhood have adverse effects disseminated across various domains of health and development. Domains predominantly affected by poor sleep in a child can be broadly grouped into behavioural, emotional, and physical health (Turnbull et al. 2013; Meltzer et al. 2014; Hysing et al. 2016). Furthermore, the family of a child with sleep problems may also be significantly impacted, thus highlighting the necessity of preventing sleep difficulties in early development (Sadeh et al. 2010). In contrast, healthy sleeping habits have been implicated in boosting the immune system and reducing the risk of cardiac disorders, hormonal disorders, and metabolic disorders (Knutson 2012; Narang et al. 2012; Srinivasan et al. 2013; Irwin 2015). The American Academy of Pediatrics recommends including sleep health as a part of anticipatory guidance throughout the development of children during routine visits to health practitioners (AAO Pediatrics 2008).

Whilst many common childhood health conditions have been associated with poor sleep (Camfferman et al. 2016; Sakamoto et al. 2017; Meltzer and Pugliese 2017; Ramirez et al. 2019), early childhood caries (ECC) has received the least attention among those. American Academy of Pediatric Dentistry (2020) has defined ECC as “the presence of one or more decayed (non-cavitated or cavitated lesions), missing (due to caries), or filled tooth surfaces in any primary tooth in a child under the age of 71 months or younger.” Despite advances in public health measures and the introduction of modern materials in the prevention of ECC, this disease has remained a significant health problem, negatively affecting the quality of life of the affected children and their families (Nora et al. 2018). ECC or severe-ECC (S-ECC) may escalate treatment costs because of frequent emergency dental and medical visits (sometimes to the extent of hospitalization), the comprehensive nature of the treatment under dental general anaesthesia (DGA), and the associated costs of school absenteeism and subsequent reduced learning ability (Nowak et al. 2014; Karki et al. 2019; Kastenbom et al. 2019). The gravity of the situation can be envisioned in the fact that the data from 188 countries have indicated the prevalence of caries in primary teeth to be 4.929205 × 10^8^ (95% CI 4.906571 × 10^8^ to 4.953852 × 10^8^) (Global Burden of Disease 2013 Collaborators 2015). S-ECC is considered a debilitating oral condition as it may impact the nutrition and, consequently, the growth of the affected child (Ayhan et al. 1996; Acs et al. 1999). The aetiology of ECC is multifactorial, and numerous risk factors have been identified that contribute to ECC initiation and progression (Kirthiga et al. 2019). One of the principal factors associated with ECC causation is the feeding of sugar-sweetened beverages and *ad libitum* feeding; hence, the condition was earlier referred to as nursing bottle caries or baby bottle decay. Some caregivers and parents of the infants prop the feeding bottle in the mouth of an infant with the intent to make them sleep, which may lead to ECC as the fermentable carbohydrates are metabolized by oral plaque biofilm (Nagarajappa et al. 2020). Apart from this indirect implication of sleep in contributing to the development of ECC, inadequate sleep also increases salivary glucose, which might alter the level of inflammatory cytokines and hence modify the disease process (Alqaderi et al. 2016). Besides, ECC or S-ECC might result in increased pain episodes in preschool children and result in frequent night-time waking episodes or sleep disturbances. Furthermore, paediatric dentists are one of the earliest healthcare providers to infants and children and might be responsible for routine care thereafter. During the routine dental care of children, and in consultation with the parent/s, paediatric dentists might also be able to identify some of the main symptoms of sleep problems, like daytime sleepiness. They are also able to help diagnose sleep problems during their routine medical history taking and examination of the anatomy of the oral soft and hard tissues and make the appropriate referral.

Although much research has been carried out to evaluate the effect of ECC on the quality of life (including sleep as one of the domains) of children, a systematic understanding of the relationship between sleep and ECC is lacking. The present systematic review aimed to investigate the effect of sleep on the development and progression of ECC. Thus, the review attempted to answer the following research question: Do sleep issues affect the occurrence or severity of early childhood caries in preschool children?

## Materials and methods

### Protocol and registration

The methodology of the current review was formulated in advance by adhering to the Cochrane Handbook and documented in the protocol (Higgins et al. 2020). Subsequently, the protocol was registered at the International Prospective Register of Systematic Reviews (Prospero protocol number CRD42020175285). The protocol was designed a priory to investigate the bidirectional relationship between sleep and ECC wherein we planned to explore the effect of sleep on ECC and vice versa; however, we could not find any study that had evaluated the effect of ECC on sleep as a primary outcome. We included only studies that had focused exclusively with sleep as the exposure and ECC as the outcome. The protocol of the review can be accessed online at: https://www.crd.york.ac.uk/prospero/display_record.php?ID=CRD42020175285. The review is being reported as per the PRISMA (Preferred Reporting Items of Systematic Reviews and Meta-analyses) statement and checklist (Moher et al. 2009).

### Study eligibility criteria

Type of studies: The studies included evaluated the effect of sleep parameters on the caries experience or severity or progression of ECC in children less than six years of age. The detailed PECO (Population, Exposure, Control, and Outcome) schema is outlined in Table 1.

**Table 1:** Eligibility criteria for the present review

Inclusion criteria	Studies evaluating the effect of sleep on early childhood caries (ECC)
Type of studies	Case–control studies, cross-sectional studies, longitudinal studies
Population (P)	Children under 6 years of age with sleep disturbances
Exposure (E)	Sleep disturbances which might include number of sleep hours, frequency of night-time waking (reported through subjective or objective parameters), bedtime routines
Control (C)	Children under 6 years of age without sleep disturbances (either a parallel-group or in intervention studies, the same children treatment of sleep disturbances served as a control)
Outcome (O)	ECC (prevalence or incidence or severity or progression of ECC)

### Information sources and literature search

Six databases were systematically searched by two independent authors (SD and EM) with no start date restrictions or language restrictions, up to and including May 22, 2020, using the broad MeSH terms and keywords. The databases searched were as follows: Web of Science, MEDLINE (via Ovid), EMBASE (via Ovid), Scopus, CINAHL, PubMed, and LILACS. Additionally, cross references of the included articles were hand-searched for any potentially relevant article meeting the eligibility criteria and the search was updated manually in August 2022 by hand searching. Grey literature was searched on www.opengrey.eu and web search for any published thesis or conference abstract. The search strategy for all the databases is presented in Appendix 1.

### Study selection

The results obtained through the search of the databases were systematically managed using Endnote X 8.2 software for Windows (Clarivate Analytics, Philadelphia, USA). After removing duplicates, two authors (SD and EM) independently screened the titles and their respective abstracts in a standardized manner to decide upon their inclusion for full-text reading as defined by the pre-set inclusion criteria. Full-text reading was also performed for the articles that did not provide clear information about the study methodology or were considered as potential articles that could have met the inclusion criteria of the review. Cohen’s kappa coefficient (*κ*) was computed to construe the level of inter-rater agreement among two reviewers after full-text reading (Cohen 1988). Any incongruity over the final inclusion was discussed among the authors, and if required, the third author (GB) acted as an arbiter.

### Data collection process

The characteristics of the individual studies and their respective statistical data were extracted individually by two authors (SD and EM) on a piloted data extraction form. Data pertaining to sleep disturbances, ECC, and its inter-relationship were extracted for the present review based on the PECO criteria defined above.

### Data items

Information and data pertaining to the following parameters were extracted from each study: author, year of study, country of study, mean age and/or range, gender distribution, the type of study, sleep disturbances identified (quantitative including the number of hours of sleep or sleep duration or qualitative parameters including night-time wakings) and methods used to assess the exposure. For outcome (i.e. ECC), data considered potentially relevant for the review were decayed-missing (or indicated to be extracted)-filled index (tooth- or surface-wise) or incidence/prevalence (caries increment for longitudinal studies) of ECC was extracted.

### Risk of bias in individual studies

The methodological quality of the included studies was assessed using the Newcastle–Ottawa Scale (NOS) for cohort studies (maximum of 9 stars) and NOS for cross-sectional studies (maximum of 10 stars) by two authors independently (SD and EM) (Wells et al. 2009). Any disagreement about the assessment was mutually discussed to achieve consensus, and an opinion from a third reviewer (GB) was sought if necessary. The tool assessed the risk of bias of studies across the following domains: selection, comparability, and outcome, and the stars were assigned if the criteria to the particular domain were met, and details can be accessed elsewhere (Wells et al. 2009).

### Summary measures and methods of analysis

The meta-analysis was planned as defined in the registered protocol but could not be performed because of the clinical heterogeneity of the included studies and different methods to assess the exposure (sleep quantity or quality).

## Results

### Study selection

**Fig. 1:**
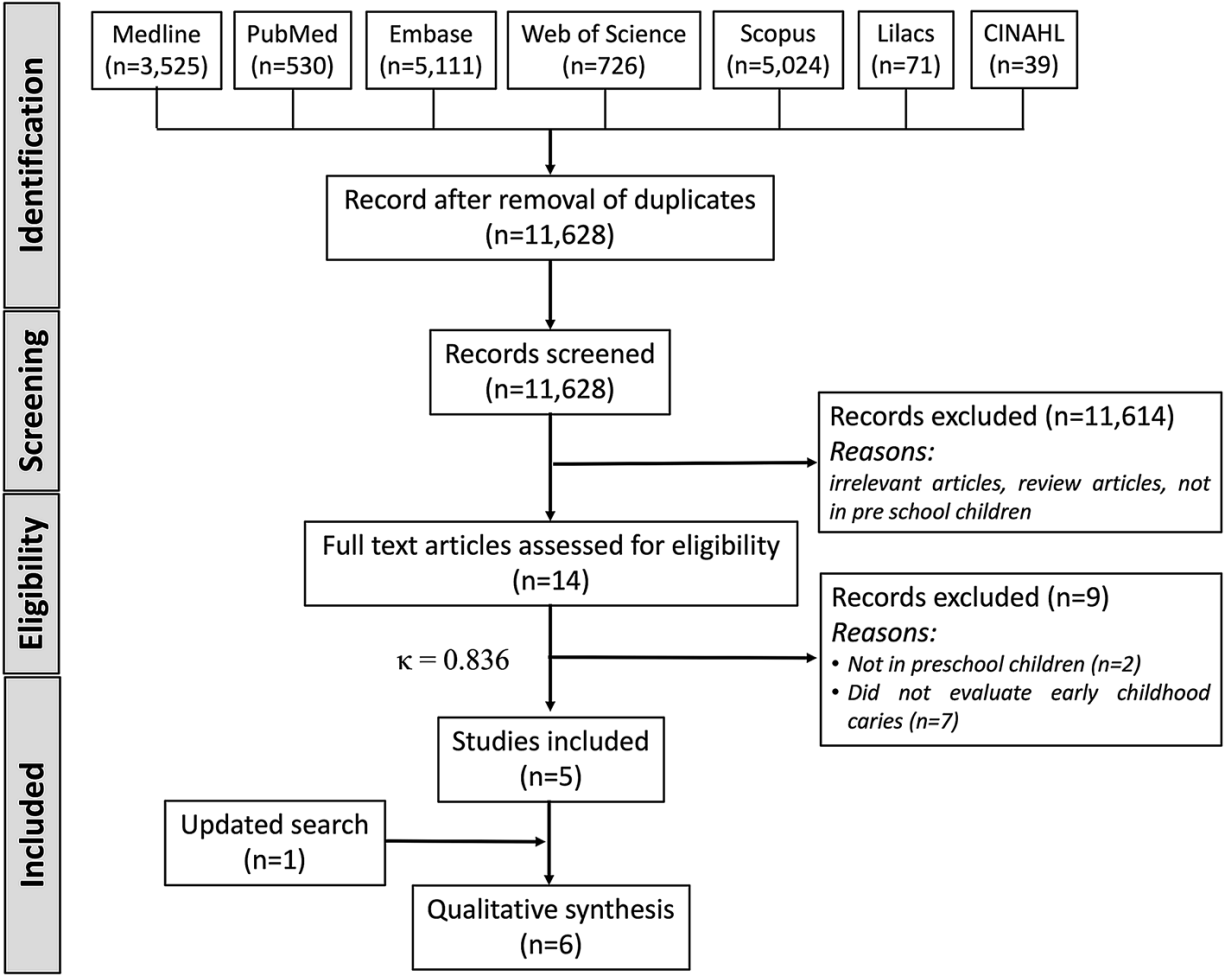
Preferred Reporting Items for Systematic Reviews and Meta-analyses flowchart used to identify studies for the effect of early childhood caries on sleep and the effect of sleep disturbances on early childhood caries

Figure 1 provides a PRISMA flow summary of the various phases in the study selection process. After electronic database searching, 15,026 records were identified, but 11,628 were screened for titles and abstracts after duplicate removal. Only 14 studies were determined to be potentially eligible for the present review and were included for full-text reading, but finally, only five were included. After updating the search in August 2022, one more cross-sectional study was added for inclusion, thus giving six studies for qualitative synthesis. The value of κ at the full-text reading stage was determined to be 0.836, indicating a good level of agreement. The details and reasons for the exclusion of nine articles after full-text reading are described in Appendix 2.

### Study characteristics and results

#### Cross-sectional studies

Four studies (Nishide et al. 2019; Zhou et al. 2019; Kitsaras e al. 2018; Ogawa et al. 2021) covering 2011 preschool children that evaluated the effect of sleep on ECC were included. All the four studies used a questionnaire to assess sleep in preschool children; the characteristics of the studies are presented in Table 2. One study (Kitsaras et al. 2018) found that children who had optimal bedtime routines had lower dmft scores compared to children with suboptimal bedtime routines (*p* = 0.011). However, the study included tooth brushing, avoidance of snacks/drinks before bed, avoidance of electronic devices before bed and book reading in the bedtime routine, apart from the regular sleeping times as one of the parameter. Besides, the number of caries lesions was significantly correlated with the mean sleep onset time (*r* = 0.370; *p* = 0.022) according to another study (Nishide et al. 2019). Sleep duration was evaluated in two studies; one (Nishide et al. 2019) did not find any significant correlation (*p* = 0.094), whereas the other study (Zhou et al. 2019) found significantly lower caries prevalence in children who sleep for longer hours. Ogawa et al. (2021) found that the sleep durations [adjusted odds ratio = 0.54 (95% CI 0.36–0.80), *p* = 0.0012] were found to be independently associated with the development of dental caries in the children in multivariable statistical analysis.

**Table 2:** Summary of cross-sectional studies that evaluated the effect of sleep on prevalence of ECC in preschool children

Author	Country	Sample size	Gender distribution	Age group	Method of assessing sleep (exposure)	Results
Kitsaras et al. (2018)	UK	50 children	Males: 48% Females: 52%	Range: 3–5 years Mean age: 4 years (SD: 0.8 months)	Interactive text survey consisting of open-ended and closed-ended questions sent to mobile phones of parents. The main independent variable was optimal bedtime routines	Children in families with optimal bedtime routines presented lesser cavities and fewer missing or filled teeth (dmft = 0) (Median = 4) compared to children in families with suboptimal bedtime routines (dmft > 0) (Grand Median = 2), *p* = 0.011
Nishide et al. (2019)	Japan	140 children (Only 38 were less than 7 years)	Males: 55% Females: 45% (gender distribution of children with primary dentition not mentioned)	Data of 38 children in primary dentition is extracted for the review	Recording form to record waking time, bedtime, mealtimes, snacking time, and tooth brushing time for 8 days at home	The number of caries was significantly correlated with the mean sleep onset time (*r* = 0.370, *p* = 0.022). No significant correlation was found between prevalence of caries and sleep duration (*p* = 0.094) and other sleep parameters
Zhou et al. (2019)	China	1591 children	Males: 51.6% Females: 48.4%	Range: 3–5 years	Structured questionnaire completed through interviews with parents or caregivers	Children who slept for 12 h or longer had lower caries prevalence. dmft scores were significantly lower (*p* < 0.05). The mean values of dmft were distributed as follows: ≥ 12 h (*n* = 130) = 3.37 ± 4.30 10–12 h (*n* = 912) = 4.24 ± 4.85 ≤ 10 h (*n* = 549) = 4.74 ± 4.72
Ogawa et al. (2021)	Japan	332 children	Males: 53.6% Females: 46.4%	Mean age: 64.4 (SD = 10.1) months; range: 3 to 6 years	Questionnaire completed through parents	There was a significant negative correlation between the sleep duration the number of caries experienced by the children (*r* = − 0.17, *p* = 0.0016). In the multivariate analysis, the sleep durations [adjusted odds ratio = 0.54 (95% CI 0.36–0.80), *p* = 0.0012] was found to be independently associated with the development of dental caries in the children

*CI* confidence intervals, *SD* standard deviation

#### Longitudinal studies

Two studies (Watanabe et al. 2014; Chen et al. 2018) recruiting 102,271 children that evaluated the effect of sleep on the occurrence of ECC were included. Both studies were conducted in Japan and had a follow-up of 18 months (Table 3). Also, the studies found that irregular bedtime was a risk factor for the development of ECC (odds ratio of 1.66–1.71), thereby indicating that children who had irregular bedtimes had a 66–71% higher chance of developing ECC (Table 3). Additionally, the risk of ECC was associated with late bedtime in a dose–response manner, and children who slept after 11 pm might have a 74–85% higher chance of developing ECC (odds ratio of 1.74–1.85). One study (Chen et al. 2018) also observed that children with shorter sleep duration had a higher chance of ECC, with children who slept less than 8 h during the night having a 30% increased risk of caries than children who slept more than 11 h.

**Table 3:** Summary of longitudinal studies that evaluated the effect of sleep on incidence of ECC in preschool children

Author	Country	Sample size	Gender distribution	Age group	Method of assessing sleep (exposure)	Results
Watanabe et al. (2014)	Japan	31,202 children at 1.5 years follow-up	Males: 51.4%; Females: 48.6%	Age at baseline: 1.5 years	Self-administered questionnaire completed by parents or guardian	Multivariate logistic regression analysis showed that a late bedtime is an independent risk factor for caries development. The OR of developing caries if the child slept between 9 and 11 pm was 1.33 (95% CI 1.23, 1.45), after 11 pm was 1.85 (95% CI 1.61, 2.12) and irregular bedtime was 1.71 (95% CI 1.51, 1.93)
Chen et al. (2018)	Japan	71,069 children	Male: 51% Female: 49%	18 months at baseline and 3 years of age at dental evaluation	Standardized parent-reported questionnaires to record child’s bedtime, wake time, and sleep duration	The risk of caries increased with bedtime becoming later in a dose–response manner, and children with irregular bedtime also had a greater risk of caries. The multivariable aORs were 1.26 (95% CI 1.19–1.33), 1.48 (1.38–1.58), 1.74 (1.58–1.92), 1.90 (1.58–2.29), and 1.66 (1.53–1.81) for bedtimes at 21:00, 22:00, 23:00, 0:00, and irregular bedtime, respectively The risk of caries was inversely proportional to sleep duration, but the associations were less pronounced than those of bedtime. Children with night-time sleep duration of ≥ 11 h had lesser caries compared to children with shorter (≤ 8 h) and irregular sleep duration (30% increased risk of caries). The multivariable aORs were 1.30 (95% CI 1.15–1.47), 1.16 (1.09–1.24), 1.11 (1.05–1.18), and 1.35 (1.25–1.46) for sleep durations of ≤ 8 h, 9 h, 10 h, and irregular sleep duration, respectively

*aOR* adjusted odds ratio, *CI* confidence interval, *OR* odds ratio

### Risk of bias within studies

#### Cross-sectional studies

Two of the included studies (Kitsaras et al. 2018; Zhou et al. 2019) received 7 stars (*******), and two others (Nishide et al. 2019, Okawa et al. 2021) received 6 stars (******) (Table 4). The studies received fewer stars in the ‘*comparability*’ domain due to the lack of control of confounding factors. In the outcome domain, the studies received less than the maximum stars because the ECC was evaluated through patient medical records rather than evaluated by the patients, which cannot estimate the caries load accurately if the teeth were extracted due to other reasons like trauma.

**Table 4:** Risk of bias of studies evaluating effect of sleep on ECC

Author (year)	Selection	Comparability	Outcome	Total stars
	Maximum 5 stars	Maximum 2 stars	Maximum 3 stars	Maximum 10 stars
(A) Cross-sectional studies				
Kitsaras et al. (2018)	****	*	**	******* (7)
Nishide et al. (2019)	***	**–**	***	****** (6)
Zhou et al. (2019)	****	*	**	******* (7)
Ogawa et al. (2021)	**	*	***	******(6)

	Maximum 4 stars	Maximum 2 stars	Maximum 3 stars	Maximum 9 stars
(B) Longitudinal studies				
Watanabe et al. (2014)	****	**	***	********* (9)
Chen et al. (2018)	****	**	***	********* (9)

#### Longitudinal studies

Both the longitudinal studies (Watanabe et al. 2014; Chen et al. 2018) that evaluated the effect of sleep on ECC were conducted on a considerable sample size with a sound methodology. Therefore, both studies received a maximum of 9 stars according to the Newcastle–Ottawa Scale for longitudinal studies (Table 4).

## Discussion

The present review originally planned to assess the bidirectional relationship between ECC and sleep disturbances in preschool children, but due to methodological constraints involving the control of confounding in study designs and clinical heterogeneity, we focussed on the unidirectional relationship exploring the role of sleep as a risk factor in ECC. Recent systematic reviews have explored various risk factors related to the causation of ECC (previous caries experience, presence of visible plaque, frequent carbohydrate consumption, poor oral hygiene, presence of enamel defects, breastfeeding, fluoride exposure) but have not considered sleep disturbance as a possible risk factor for ECC (Kirthiga et al. 2019; Moynihan et al. 2019). Thus, the present systematic review contributes significantly to exploring the effect of sleep disturbance as a potential risk factor for ECC. Furthermore, several systematic reviews have addressed the impact of ECC on the OHRQoL of preschool children or the improvement in OHRQoL after rehabilitation of children with ECC; however, these reviews do not emphasize the importance of sleep in the causation of ECC (Jankauskiene and Narbutaite 2010; Nora et al. 2018).

Untreated S-ECC is a debilitating oral condition and may cause dental pain, abscess, and cellulitis in preschool children (Ferraz et al. 2014). Hence, it is perhaps no surprise that the results obtained from the present systematic review highlight that ECC likely contributes to disturbed sleep in preschool children. The precise mechanism as to how ECC affects sleep is likely via dental pain, disturbing the sleep of children. However, the mechanisms by which sleep disturbance may cause ECC still require to be elucidated and will require a plethora of studies to explore various intermediate complex steps. The pathogenesis for ECC is the conversion of fermentable carbohydrates on teeth to the metabolic end-products by the action of microbes in the plaque biofilm (mainly *Streptococcus mutans* aided by *Lactobacilli*) which manifests as demineralization of the hard tissues of the teeth (enamel and dentine). Healthy sleeping habits are known to enhance the immunity of an individual against pathogenic organisms (Bryant et al. 2004; Bollinger et al. 2009), and therefore, it seems logical that inadequate sleep predisposes an individual to dental caries by altering the immune status and predisposing them to carious activity by *Streptococcus mutans*.

Additionally, sleep disturbances are associated with hormonal and metabolic disturbances and have even been thought to be related to salivary glucose levels and the causation of gingival inflammation (Leproult and Van Cauter 2010; Kirthiga et al. 2019). Carbohydrates such as sucrose, fructose, and glucose are known substrates for *Streptococci mutans*, and the intricate mechanism between sleep, salivary glucose, and caries should be explored further. Shorter sleep duration or delayed bedtime is also associated with reduced self-regulation of appetite in children and may be related to over-eating behaviours (Burt et al. 2014; Arun et al. 2016; Miller et al. 2019). This consideration might have significant importance for caregivers, healthcare providers, and future researchers because ad libitum bottle feeding and breastfeeding by mothers to get their children to sleep is a proven risk factor for ECC (Avila et al. 2015; Feldens et al. 2018). Additionally, as excessive daytime sleepiness in children is often manifested in problems with behaviour, children might have difficulty brushing or letting their parents brush their teeth, thereby aggravating the chances of ECC. Thus, interventions towards improving the sleep of children and educating mothers about healthy sleeping habits may enhance timely weaning and regulation of eating behaviours in children and potentially prevent ECC.

### Causal versus association relationship

**Fig. 2:**
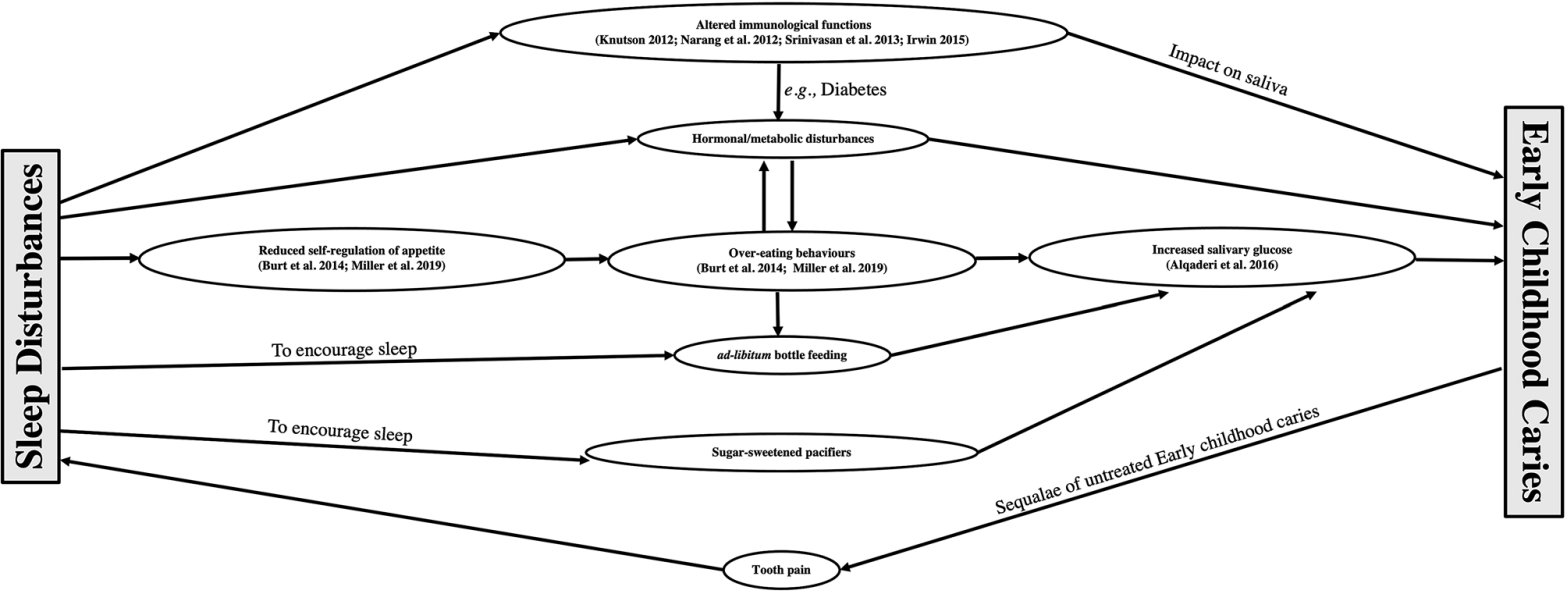
Conceptual model diagram demonstrating potential pathways of association between sleep and early childhood caries

The findings from the present systematic review were obtained from two well-conducted large longitudinal studies and four cross-sectional studies. However, the causal relationship between sleep (presumed cause) and ECC (observed effect) cannot be established conclusively as it might not follow Hill’s criteria for causation (which include strength, consistency, specificity, temporality, biological gradient, plausibility, coherence, experiment, and analogy) (Hill 1965). Nevertheless, sleep quantity and quality do have an association with ECC and, based on the results of the review, might be independent risk factors for ECC. The confounding structure and the various mediators that might be involved in the association between ECC and sleep are depicted in Fig. 2.

### Strengths and limitations

An extensive search of databases (as evident in the number of records identified), prior registration of review protocol at the International Prospective Register of Systematic Reviews, and strict eligibility criteria for the inclusion/exclusion of the articles are some of the strengths of the present systematic review. The main limitation of using patient-reported outcomes to measure sleep is that it might be subjective and vulnerable to many biases that are commonly associated with questionnaires (*e.g.* response bias, recall bias, demand characteristics, etc.). Thus, parents might overemphasize their child’s sleep disturbances and misconstrue other sleep parameters (like night waking) as trivial (Holley et al. 2010; Nelson et al. 2014). Although we planned to include other criteria for measuring sleep in children with ECC, surprisingly, we could not find any study that had used an objective means to measure the quantity or quality of sleep or evaluated sleep precisely as a primary outcome. The main limitation of using patient-reported outcomes to measure sleep is that it might be subjective and vulnerable to many biases that are commonly associated with questionnaires (e.g. response bias, recall bias, demand characteristics, etc.). Thus, parents might overemphasize their child’s sleep disturbances and misconstrue other sleep parameters (like night waking) as trivial (Holley et al. 2010; Nelson et al. 2014). Even though we found well-conducted studies with a large sample that evaluated the effect of poor sleep as a risk factor for ECC, these studies also suffer the same drawback because of the lack of objective criteria in the measurement of the sleep.

### Future research and recommendations

Future studies should aim at evaluating the effect of sleep on ECC or vice versa using some objective tool for assessing sleep like polysomnography or actigraphy (Beck and Marcus 2009; Smith et al. 2018). However, polysomnography measures only one night of sleep and is invasive, which might render it less beneficial to measure the relationship between sleep and caries, which is considered a chronic infectious disease. On the other hand, actigraphy assesses both sleep quantity and quality and is best for measuring habitual sleep patterns, usually for up to 7 days; thus, it could be used to explore the bidirectional relationship between ECC and sleep. Sleep-disordered breathing (SDB) is a well-known condition with a reported prevalence of around 4–11% in children, with the more severe form, obstructive sleep apnoea (OSA), occurring in 1–4% (Lumeng and Chervin 2008). No studies covering this condition were identified in the initial search. Thus, SDB/OSA might have been a confounder for the development of ECC, particularly since mouth breathing is often a feature of OSA. We suggest it would be helpful for future studies assessing the relationship between sleep issues and ECC to include an evaluation of SDB and OSA. The paediatric dentist can view tonsillar enlargement and tonsillar disease as potentially contributing to OSA and sleep issues in children and suggest the appropriate referral. Future research could also examine the links between sleep and ECC and the underlying mechanisms of the relationship between two of these common conditions. Also, it is recommended to conduct case–control studies and control confounding factors to identify any further causal relationship between sleep and ECC.

## Conclusions

To conclude, sleep disturbance (late or irregular bedtimes and fewer sleeping hours) was found to be a risk factor for ECC. The risk of ECC might be related inversely in a dose–response manner to the number of sleep hours. Clinically, the results of the present review might be relevant as they may help in guiding paediatric dentists to educate parents about the importance of regular sleeping time and a good amount of sleeping hours to prevent ECC in preschool children.

## Appendix 1: Search strategy

**Ovid Medline** Ovid MEDLINE(R) and Epub Ahead of Print, In-Process & Other Non-Indexed Citations, Daily and Versions(R) 1946 to May 20, 2020.

**Searched on** 21 May 2020.

**Table:**

S no.	Search keyword	Number of yields
1	exp Sleep/ or sleep.mp. or exp Sleep Deprivation/ or exp Sleep Hygiene/ or exp Sleep Wake Disorders/ or exp Sleep Medicine Specialty/	200,710
2	Sleep duration	7671
3	exp "Sleep Initiation and Maintenance Disorders"/ or disturbed sleep.mp	14,726
4	Sleep fragmentation.mp	1590
5	Behavioural insomnia.mp	7
6	Bedtime resistance.mp	134
7	Waking after sleep.mp	44
8	waking during sleep.mp	5
9	quality of life.mp. or exp "Quality of Life"/	340,174
10	1 or 2 or 3 or 4 or 5 or 6 or 7 or 8 or 9	527,275
11	dental caries.mp. or exp Dental Caries/	51,562
12	toothache.mp. or exp Toothache/	3509
13	oral swelling.mp	45
14	oral health.mp. or exp Oral Health/	31,541
15	acute pain.mp. or exp Pain/ or exp Acute Pain/	395,524
16	early childhood caries.mp	1484
17	11 or 12 or 13 or 14 or 15 or 16	470,224
18	child.mp. or exp Child, Preschool/ or exp Child/ or exp Child Health/	2,087,472
19	exp Infant/ or infant.mp. or exp Infant Care/	1,189,263
20	(child* or preschool* or pre-school* or kindergarten* or p?ediatric*).mp	2,510,052
21	18 or 19 or 20	2,980,854
21	10 and 17 and 21	3525

**Embase: Embase classic + Embase** 1997 to 2020 May 20.

**Searched on** 21 May 2020.

Results: 5111

**Table:**

S no.	Search keyword	Number of yields
1	exp Sleep/ or sleep.mp. or exp Sleep Deprivation/ or exp Sleep Hygiene/ or exp Sleep Wake Disorders/ or exp Sleep Medicine Specialty/	423918
2	Sleep duration.mp. or exp sleep time/	31217
3	exp sleep disorder/ or disturbed sleep.mp	241168
4	Sleep fragmentation.mp.	2929
5	bedtime resistance.mp.	263
6	quality of life.mp. or exp "quality of life"/	587786
7	1 or 2 or 3 or 4 or 5 or 6	975092
8	dental caries.mp. or exp dental caries/	60080
9	caries.mp.	66332
10	exp pain/ or exp chronic pain/ or exp jaw pain/ or pain.mp. or exp face pain/	1647841
11	(mouth or tooth or teeth or oral).mp. [mp = title, abstract, heading word, drug trade name, original title, device manufacturer, drug manufacturer, device trade name, keyword, floating subheading word, candidate term word]	2066552
12	10 and 11	230781
13	8 or 9 or 12	294585
14	(child* or preschool* or pre-school* or kindergarten* or p?ediatric*).mp. [mp = title, abstract, heading word, drug trade name, original title, device manufacturer, drug manufacturer, device trade name, keyword, floating subheading word, candidate term word]	3123213
15	exp preschool child/ or exp child/ or child.mp	3228642
16	exp infant/ or infant.mp.	1242912
17	14 or 15 or 16	3774428
18	7 and 13 and 17	5111

Web of Science

**Searched on** 21 May 2020.

**Results**: 726

# 4#3 AND #2 AND #1 Results: 726.

# 3TS=((child*) OR (infant*) OR (preschool*) OR (pre-school*) OR (kindergarten*) OR (p?ediatric*)) Results: 2,165,015

# 2TS=((Dental caries) OR (caries) OR (toothache) OR (tooth pain)OR (oral swelling) OR (oral health) OR (bottle feeding) OR (dental care for children) OR (early childhood caries) OR (acute pain) OR (facial pain) OR(face pain)) Results: 199,288

# 1TS=((Sleep) OR (Sleep Deprivation) OR (sleep duration) OR (sleep problem) OR (disturbed sleep) OR (sleep fragmentation) OR (Sleep Wake Disorders) OR (waking after sleep onset) OR (behavioural insomnia) OR (bedtime resistance) OR (Sleep Medicine Specialty)) Results: 238,585

**Lilacs**

**Searched on** 21 May 2020.

**Results**: 71

((sleep) OR (sleep deprivation) OR (sleep problem) OR (disturbed sleep) OR (sleep fragmentation) OR (sleep wake disorders) OR (waking after sleep onset) OR (behavioural insomnia) OR (bedtime resistance) OR (sleep medicine specialty)) AND ((dental caries) OR (caries) OR (toothache) OR (tooth pain) OR (oral swelling) OR (oral health) OR (bottle feeding) OR (dental care for children) OR (early childhood caries) OR (acute pain) OR (facial pain) OR (face pain)) AND ((child*) OR (infant*) OR (preschool*) OR (pre-school*) OR (kindergarten*) OR (p?ediatric*)).

**Scopus**

**Searched on** 21 May 2020.

**Results**: 5024

( ( TITLE-ABS-KEY ( sleep)) OR ( TITLE-ABS-KEY ( sleep AND deprivation)) OR ( TITLE-ABS-KEY ( sleep AND duration)) OR ( TITLE-ABS-KEY ( insomnia)) OR ( TITLE-ABS-KEY ( ( oral AND ( quality AND of AND life))))) AND ( ( ( dental AND ( caries)) OR ( toothache) OR ( ( tooth OR dental OR mouth) AND pain) OR ( ( oral OR mouth OR facial) AND swelling))) AND ( ( child* OR p?diatric OR infant OR preschool* OR pre-school*)).

**PubMed**

**Searched on** 21 May 2020.

**Results**: 530

((((((((((((((((((((((sleep) OR ("sleep deprivation")) OR ("sleep duration")) OR ("sleep problem")) OR ("disturbed sleep")) OR ("sleep fragmentation")) OR ("waking after sleep onset")) OR ("behavioural insomnia")) OR ("bedtime resistance"))) OR (((("Sleep"[Mesh]) OR "Sleep Deprivation"[Mesh]) OR "Sleep Wake Disorders"[Mesh]) OR "Sleep Medicine Specialty"[Mesh]))) AND ((((((((("Dental Caries"[Mesh:NoExp]) OR "Toothache"[Mesh]) OR "Oral Health"[Mesh]) OR "Bottle Feeding"[Mesh]) OR "Dental Care for Children"[Mesh]) OR "Acute Pain"[Mesh]) OR "Facial Pain"[Mesh])) OR (((((((caries) OR (toothache) OR ("tooth pain")) OR ("oral swelling")) OR ("oral health")) OR ("bottle feeding")) OR ("early childhood caries")) OR ("acute pain")) OR ("facial pain")) OR ("face pain"))) AND ((((("Child"[Mesh]) OR "Infant"[Mesh]))) OR ((child* OR girl* OR boy* OR preschool* OR pre-school* OR kindergarten* OR p?ediatric*))))))))).

**CINAHL plus**

**Searched on** 21 May 2020.

**Results**: 39

((sleep) OR (sleep deprivation) OR (sleep problem) OR (disturbed sleep) OR (sleep fragmentation) OR (sleep wake disorders) OR (waking after sleep onset) OR (behavioural insomnia) OR (bedtime resistance) OR (sleep medicine specialty)) AND ((dental caries) OR (caries) OR (toothache) OR (tooth pain) OR (oral swelling) OR (oral health) OR (bottle feeding) OR (dental care for children) OR (early childhood caries) OR (acute pain) OR (facial pain) OR (face pain)) AND ((child*) OR (infant*) OR (preschool*) OR (pre-school*) OR (kindergarten*) OR (p?ediatric*)).

**Grey literature search:** on www.opengrey.eu and first ten pages of Google using the broad keywords (Sleep and Caries, or sleep and dental, or sleep and early childhood caries).

**Results**: 0

## Reasons for articles excluded after full-text reading (*n* = 140)

**Table:**

S. no.	Study ID	Reason for exclusion
1	Aktürk Z, Dönmez H, Güçlü M, Koru C. Health screening among Riyadh International Turkish School students: Prominent problems are dental caries and irregular sleep. *Turkiye Klinikleri Journal of Medical Sciences*. 2010;30(3):940–946	Not in preschool children
2	Amaral A, Melao N. Health profile of children monitored in primary care consultation in Viseu, Portugal. [Portuguese]. *Revista Portuguesa de Saude Publica*. 2016;34(1):53–60	Not in preschool children
3	Arvidsson L, Birkhed D, Hunsberger M, Lanfer A, Lissner L, Mehlig K, Mårild S, Eiben G. BMI, eating habits and sleep in relation to salivary counts of mutans streptococci in children–the IDEFICS Sweden study. *Public Health Nutrition.* 2016;19(6):1088–92	Did not evaluate early childhood caries
4	Asgari I, Kazemi E. Cross-cultural adaptation of Persian version of scale of oral health outcomes for 5-year-old children. *Journal of Dentistry / Tehran University of Medical Sciences.* 2017;14(1):48–54	Did not evaluate early childhood caries
5	Bordoni N, Ciaravino O, Zambrano O, Villena R, Beltran-Aguilar E, Squassi A. Early childhood oral health impact scale (ECOHIS): translation and validation in Spanish language. *Acta Odontológica Latinoamericana.* 2012;25(3):270–8	Did not evaluate early childhood caries
6	Born CD, Divaris K, Zeldin LP, Rozier RG. Influences on preschool children's oral health-related quality of life as reported by English and Spanish-speaking parents and caregivers. *Journal of Public Health Dentistry*. 2016;76(4):276–286	Did not evaluate early childhood caries
7	Broder HL, McGrath C, Cisneros GJ. Questionnaire development: face validity and item impact testing of the Child Oral Health Impact Profile. *Community Dentistry & Oral Epidemiology*. 2007;35 Suppl 1:8–19	Did not evaluate early childhood caries
8	Burgette JM, Preisser JS, Weinberger M, King RS, Lee JY, Rozier RG. Enrollment in early head start and oral health-related quality of life. *Quality of Life Research*. 2017;26(10):2607–2618	Did not evaluate early childhood caries
9	Karki S, Pakkila J, Laitala ML, Humagain M, Anttonen V. Influence of dental caries on oral health-related quality of life, school absenteeism and school performance among Nepalese schoolchildren. *Community Dentistry & Oral Epidemiology*. 2019;47(6):461–469	Did not evaluate early childhood caries
